# From Listing to Recovery: A Review of Nutritional Status Assessment and Management in Liver Transplant Patients

**DOI:** 10.3390/nu15122778

**Published:** 2023-06-16

**Authors:** Federico Ravaioli, Nicola De Maria, Lorenza Di Marco, Alessandra Pivetti, Riccardo Casciola, Carlo Ceraso, Gabriella Frassanito, Martina Pambianco, Maddalena Pecchini, Chiara Sicuro, Laura Leoni, Stefano Di Sandro, Paolo Magistri, Renata Menozzi, Fabrizio Di Benedetto, Antonio Colecchia

**Affiliations:** 1 Gastroenterology Unit, Department of Medical Specialties, University Hospital of Modena, University of Modena & Reggio Emilia, 41121 Modena, Italy; f.ravaioli@unibo.it (F.R.); demaria.nicola@aou.mo.it (N.D.M.); lor.dimarco@gmail.com (L.D.M.); pivetti.alessandra@aou.mo.it (A.P.); riccardocasciola@gmail.com (R.C.); cerasocarlo@gmail.com (C.C.); gabriellafrassanito@gmail.com (G.F.); pambiancomartina@gmail.com (M.P.); madpecchini@gmail.com (M.P.); chiarasicuro44@gmail.com (C.S.); 2 Clinical and Experimental Medicine PhD Program, University of Modena and Reggio Emilia, 41121 Modena, Italy; 3 Division of Metabolic Diseases and Clinical Nutrition, Department of Specialistic Medicines, University Hospital of Modena and Reggio Emilia, Largo del Pozzo 71, 41125 Modena, Italy; leoni_laura@hotmail.it (L.L.); renata.menozzi@unimore.it (R.M.); 4 Hepato-Pancreato-Biliary Surgery and Liver Transplantation Unit, University Hospital of Modena “Policlinico”, University of Modena and Reggio Emilia, 41121 Modena, Italy; stefano.disandro@unimore.it (S.D.S.); paolo.magistri@unimore.it (P.M.); fabrizio.dibenedetto@unimore.it (F.D.B.)

**Keywords:** chronic liver disease, malnutrition, nutrition, ACLD, advanced liver diseases, liver transplantation, cirrhosis, bariatric surgery, transplant, sarcopenia

## Abstract

Liver transplantation (LT) is a complex surgical procedure requiring thorough pre- and post-operative planning and care. The nutritional status of the patient before, during, and after LT is crucial to surgical success and long-term prognosis. This review aims to assess nutritional status assessment and management before, during, and after LT, with a focus on patients who have undergone bariatric surgery. We performed a comprehensive topic search on MEDLINE, Ovid, In-Process, Cochrane Library, EMBASE, and PubMed up to March 2023. It identifies key factors influencing the nutritional status of liver transplant patients, such as pre-existing malnutrition, the type and severity of liver disease, comorbidities, and immunosuppressive medications. The review highlights the importance of pre-operative nutritional assessment and intervention, close nutritional status monitoring, individualised nutrition care plans, and ongoing nutritional support and monitoring after LT. The review concludes by examining the effect of bariatric surgery on the nutritional status of liver transplant recipients. The review offers valuable insights into the challenges and opportunities for optimising nutritional status before, during, and after LT.

## 1. Introduction

The relation between the nutritional status of patients with advanced chronic liver disease (cirrhosis) and clinical outcomes has become increasingly evident in the last few years. In advanced chronic liver disease (ACLD), when the liver reaches a point where it can no longer regenerate and repair itself effectively, liver transplant (LT) emerges as the definitive treatment option [1]. The current review aims to summarise the role and the importance of an accurate assessment of the nutritional status of the cirrhotic patient undergoing LT, as well as the management of nutrition before, during, and after LT in order to improve long-term clinical outcomes.

## 2. Data Sources and Searches

We conducted a comprehensive search for English language publications related to our research topic on various databases, including MEDLINE, Ovid, In-Process, Cochrane Library, EMBASE, and PubMed, up until March 2023. Our literature searches were conducted using specific keywords to ensure that we obtained all relevant information on our topic: nutrition, malnutrition, diet, dietitian, LT, orthotopic liver transplant, supplementation, liver diseases, chronic liver disease, advanced chronic liver disease, cirrhosis, sarcopenia, sarcopenic, muscle depletion, and muscle mass.

## 3. Malnutrition and Sarcopenia in Cirrhotic Patients

Malnutrition is a term used to describe a nutritional disorder resulting from inadequate nutrient intake that causes an alteration in body composition [2].

The prevalence of malnutrition in patients with cirrhosis ranges from 72% in alcoholics, according to the study by Franco et al. and Maharshi et al. [3,4], to 30% in cirrhosis of other etiologies, as reported by Merli et al. [5]. Merli showed in another study [6] that malnutrition is higher in males (severely malnourished M: F = 11.4%: 6.8%). In contrast, McCullough [7] reports the experiences of various authors who did not find differences between alcoholic and non-alcoholic etiologies.

The percentage of malnutrition increases proportionally with the degree of hepatic impairment, expressed by the Child–Turcotte–Pugh score (CTP), as shown by Carvalho, who reports malnutrition rates of 46% in patients with CTP class A, 84% in class B, and 95% in class C [8]. The study by Merli et al. shows that malnutrition reduces the survival of patients with CTP class A and B, while it is less related to survival in the case of more advanced stages of liver disease (CTP class C) [5].

Sarcopenia identifies a loss of muscle mass and function due to age or acute or chronic diseases, including cirrhosis; it is a significant component of malnutrition and a persistent complication in cirrhosis, negatively impacting survival, quality of life, and survival after LT [2,9].

The proportion of cirrhotic patients presenting sarcopenia ranges from 30 to 70%, depending on the diagnostic methods used and their cut-offs, gender, age, ethnicity, degree of hepatic impairment, and presence of hepatocellular carcinoma [10,11,12,13]. The gold standard for diagnosing sarcopenia is measuring skeletal muscle mass on cross-sectional imaging, and it can be obtained through various techniques such as the calculation of the psoas or the dorsal muscle area and the skeletal muscle index (SMI) [14].

In 2016, the Global Leadership Initiative on Malnutrition (GLIM) stated that a combination of phenotypical and etiological criteria are required to diagnose malnutrition, including reduced nutrient intake in the presence of acute or chronic inflammatory diseases, weight loss, or reduction in muscle mass [15]. The important role of malnutrition and sarcopenia in cirrhosis is due to the high prevalence and development of complications (hepatic encephalopathy, infections, and ascites); moreover, they affect overall clinical outcomes, including pre-and post-transplant survival and quality of life [16,17].

The pathogenesis of malnutrition and sarcopenia is complex and multifactorial. At the base of malnutrition, there is a reduced intake of macronutrients due to anorexia or a sense of early fullness, nausea, and dysgeusia. Generally, cirrhotic patients have limited knowledge of their disease self-management, which leads them to reduce or increase their food intake. In addition, there is also an impaired intake of micronutrients due to malabsorption, especially of folate, thiamine, magnesium, zinc, vitamin D, and other fat-soluble proteins [18,19,20]. In cirrhotic patients, altering the catabolic state leads to an imbalance between the needed and taken energies. The state of chronic inflammation is typical of liver cirrhosis and promotes the development and onset of sarcopenia and related complications through reduced protein synthesis and increased protein degradation. The altered protein metabolism, particularly of branched-chain amino acids (BCAAs), which are essential to support glutamine synthesis and detoxification of extrahepatic ammonia, reduces circulating BCAA levels and consequently increases muscle consumption [21,22]. Furthermore, the increase of circulating ammonia, due to reduced detoxification capacity in combination with the presence of portosystemic shunts, has pathological effects on the muscles [23,24]; ammonia is myotoxic through mechanisms including decreased protein synthesis, increased autophagy, proteolysis, and mitochondrial oxidative function in skeletal muscle. In particular, liver cirrhotic patients with sarcopenia are at high risk for developing carnitine deficiency and, therefore, should evaluate whether supplementation might be an important strategy [25]. Moreover, the alteration of metabolism results from a reduced hepatic synthesis and reserve of glycogen, an early shift from glycogenolysis to gluconeogenesis, the oxidation of fatty acids, and increased protein degradation [26,27]. Other factors related to cirrhosis aetiology that can contribute to malabsorption are the presence of portosystemic shunts, pancreatic enzyme deficiency, enteropathy, and alterations of the intestinal bacterial flora [28,29].

The etiology of liver disease, illness duration, and other co-morbidities contribute to the severity of sarcopenia [17]. The prevalence of sarcopenia varies depending on the etiology of cirrhosis and staging of liver disease [30]; in alcoholic cirrhosis, there is a higher rate of sarcopenia, which reaches up to 80% in the case of decompensated cirrhosis. Moreover, in alcohol-related cirrhosis, sarcopenia occurs more rapidly than in other etiologies since exposure to alcohol increases muscle autophagy and inhibits proteasome activity [31,32]. In NASH, viral, and autoimmune cirrhosis, sarcopenia rates of approximately 60% are reported [33]. In NASH cirrhosis, sarcopenia appears more linked to insulin resistance and chronic inflammation [34]. Cholestasis involves an alteration of the enterohepatic circulation of bile salts, and this causes high (and potentially toxic) levels of bile salts in the blood, altered metabolism and absorption of long-chain fatty acids, and a deficit of fat-soluble vitamins. In addition, it has been observed in a recent experimental study that high levels of bile acid, Colic Acid, and Deoxycholic Acid can lead to the atrophy of skeletal muscles through the G protein-coupled bile acid receptor 1 (or TGR5) that is expressed in healthy muscles [35].

Other determinants in the etiopathogenesis of malnutrition and sarcopenia are a sedentary lifestyle [36], inactivity [36], and social determinants of health [37]. A sedentary lifestyle and inactivity lead to obesity. Following the increasing trend of obesity incidence worldwide, obesity in cirrhotic patients has also increased and is estimated to occur in about 33% of liver transplant recipients in the USA [38,39,40]. The diagnosis of obesity is based on a calculation of body mass index, with a cut-off of ≥30 kg/m^2^ in Caucasian populations and ≥25 kg/m^2^ in Asian populations; however, in cirrhotic patients, the calculation is more complex for fluid retention.

The combination of obesity and sarcopenia is defined as “sarcopenic obesity” (SO) [41,42,43]. The term SO was first introduced by Baumgartner et al. to describe the condition of obesity and sarcopenia in a court of elderly and healthy patients [44].

**Figure 1:**
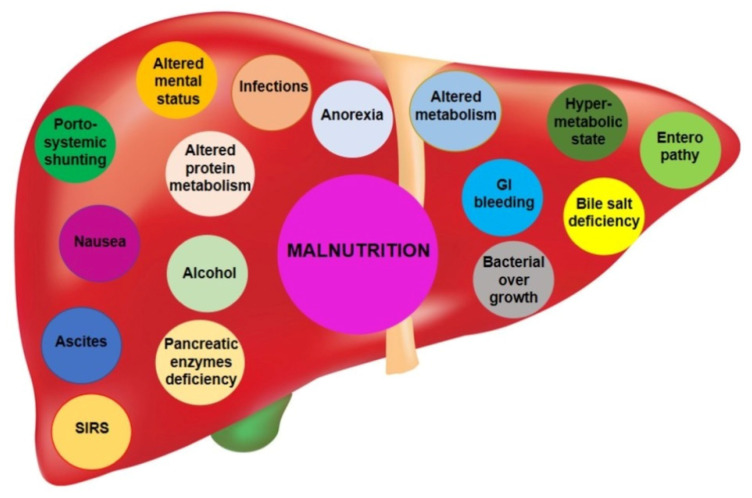
Pathophysiological mechanisms related to malnutrition in cirrhosis.

Existing studies on SO in cirrhotic patients are limited, but from existing data, we can estimate the prevalence to be around 20–35%, with a 1.5-fold increase in mortality compared to cirrhotic patients without SO [45], and this seems largely due to a higher frequency of sepsis [45]. Prompt diagnosis and correction of malnutrition in patients with cirrhosis are critical to improving outcomes [33]. The graphical representation in Figure 1 illustrates the various pathophysiological mechanisms and conditions that contribute to the development of malnutrition in patients with cirrhosis.

## 4. Nutritional Screening and Assessment in Cirrhosis

Most recent guidelines have emphasised the relevance of malnutrition screening in patients with liver cirrhosis [2,33]. However, even if there are different tools to define nutritional status, this is challenging in clinical practice, and most tools still need to be validated in cirrhotic patients.

### 4.1. Nutritional Screening Tools

The process of nutritional screening is essential in the identification of individuals who may be malnourished or at risk of malnutrition. This screening is conducted to determine the need for a comprehensive nutritional assessment. It is important to note that the definition of nutritional screening remains consistent across both the American Society of Parenteral and Enteral Nutrition (ASPEN) [46] and the European Society for Clinical Nutrition and Metabolism (ESPEN) [47]. Nutritional risk detection tools are used daily to detect potential or manifested malnutrition. These tools should be sensitive, specific, reproducible, quick to use, standardised, and validated. Screening methods must include at least three features: unintentional weight loss, unfitted nutrition, individual’s functional capacity, and disease-associated metabolic stress. Among the most used, we list the following (Table 1):

- The Mini Nutritional Assessment Short Form (MNA-SF) is a concise version of the MNA that comprises only six crucial components, with the highest level of sensitivity and specificity in comparison to the full version of the MNA and the standard nutritional assessment;
- The Malnutrition Universal Screening Test (MUST) classifies patients into malnutrition risk grades based on BMI and history of involuntary weight loss, which can also be secondary to acute illness;
- The Simplified Nutritional Appetite Questionnaire (SNAQ) is a succinct assessment comprising three inquiries pertaining to weight loss (exceeding 6 kg in the past six months or 3 kg in the past month), decreased appetite, and the necessity for nutritional supplementation within the last month;
- The Nutritional Risk Screening 2002 tool (identified by plate number 1) assesses the nutritional status and disease severity of patients over 70 years old. It has been extensively studied and validated in randomized controlled trials, proving its reliability.
- The Malnutrition Screening Tool (MST) is a quick and easy screening tool that includes questions about appetite, nutritional intake, and recent weight loss;
- The Nutrition Risk in the Critically Ill (NUTRIC Score) includes the absence of food intake, whether acute or chronic, inflammation, nutritional status, and outcomes;
- The Nutritional Risk Index (NRI) is a highly effective screening tool that was initially introduced by Buzby et al. [48] to examine the correlation between malnutrition and surgical outcomes.

**Table 1:** Nutritional screening tools to assess the risk of malnutrition in patients and specific chronic liver diseases.

Nutritional Screening Tool	Variables Included	Pro	Cons
*Mini Nutritional Assessment Short Form (MNA-SF)* [49]	Decrease in food intake Weight loss Mobility Psychological stress/acute disease Neuropsychological problems BMI	Predictive validity for adverse outcome, social functioning, mortality Practical Greatest sensitivity and specificity compared to the full form of the MNA	Interrater reliability modest Weight from fluid collections (ascites, peripheral edema) not accounted for Disease severity not considered
*Malnutrition Universal Screening Test (MUST)* [50]	Unplanned weight loss in past 3–6 months Acutely ill and unable to eat for > 5 days BMI	High interrater reliability Content and predictive validity for length of hospital stay and mortality Practical	Weight from fluid collections (ascites, peripheral edema) not accounted for Disease severity not considered
*Simplified Nutritional Appetite Questionnaire (SNAQ)* [51]	Unintentional weight loss Decreased appetite Use of supplements or tube feeding	Practical Facilitates identification and treatment of malnourished inpatients	Weight from fluid collections (ascites, peripheral edema) not accounted for Disease severity not considered
*Nutritional Risk Screening 2002 (NRS 2002)* [52]	Weight loss Food intake BMI Disease severity	Content and predictive validity Moderately reliable Practical Considers disease severity	Weight from fluid collections (ascites, peripheral edema) not accounted for
*Malnutrition Screening Tool (MST)* [53]	Unintentional weight loss Quantity of weight lost Decreased appetite	Simple/practical Predictive validity for length of stay Good reliability Highly sensitive	Weight from fluid collections (ascites, peripheral edema) not accounted for Disease severity not considered
*Nutrition Risk in the Critically Ill (NUTRIC Score)* [53,54]	Absence of food intake, whether acute or chronic Age APACHE II and SOFA scores Comorbidities Days in hospital pre-ICU Interleukin-6	Externally validated	Interleukin-6 not widely available Requires training Classic nutrition parameters not considered
*Nutritional Risk Index (NRI)* [55]	Albumin Weight loss	Simple Facilitates identification of malnourished inpatients	Weight from fluid collections (ascites, peripheral edema) not accounted Disease severity not considered
Liver disease-tailored			
*The Royal Free Hospital-Nutritional Prioritizing Tool (RFH-NPT)* [56]	Alcoholic hepatitis or tube feeding Considers fluid overload Dietary intake reduction Weight loss One option for assessing diuretic use	Simple/practical cirrhosis-specific features Excellent intraobserver and interobserver reproducibility Good external validity Predictive of clinical deterioration and transplant-free survival	Valid in population with cirrhosis only Impact of nutritional therapy based on screening score unknown
*The Liver Disease Undernutrition Screening Tool (LDUST)* [57]	Nutrient intake Weight loss Subcutaneous fat loss Muscle mass loss Fluid accumulation Decline in functional status	Quick and easily Detecting undernutrition in both inpatients and outpatients Weight from fluid collections (ascites, peripheral edema) accounted for	Relies on the patient’s subjective judgment A negative screen was unable to reliably rule out undernutrition

Different screening methods are recommended for different patient groups. According to ESPEN, for hospitalized patients, use NRS-2002; for community assessment, use MUST screening; and for elderly patients, use the first part of MNA-SF. Each method has been proven reliable and valid for its intended group [58]. None of the recognised nutrition screening tools has been validated in the setting of liver disease; for this reason, cirrhosis-specific tools have been developed in recent years. The EASL guidelines on nutritional assessment and management in patients with chronic liver disease [2] identify two criteria that can stratify patients at high risk of malnutrition: (i) being underweight, defined as a body mass index (BMI) < 18.5 kg/m^2^ [59], and (ii) having decompensated advanced chronic liver diseases (dACLD, Child–Pugh C patients) [60].

### 4.2. Specific Nutritional Assessment for Liver Disease

As a specific tool for cirrhosis, The Royal Free Hospital-Nutritional Prioritizing Tool (RFH-NPT) was developed through validation against the Royal Free Hospital SGA. It takes a mere 3 min to complete, classifying patients into low, medium, and high-risk categories based on the variables of alcoholic hepatitis, fluid overload, BMI, unplanned weight loss, and reduced dietary intake. Significantly, the Malnutrition Universal Screening Tool, which is the preferred screening tool for outpatients recommended by ESPEN, is integrated into the RFH-NPT for patients who do not have fluid overload. The content validity of this tool has shown promise in a population with cirrhosis and encompasses features of clinical and metabolic risk, along with classical nutritional variables that may influence responses to nutrition therapy. Furthermore, although BMI is a variable in the tool, it is only considered in the absence of fluid overload. The nutritional prioritizing tool utilized by the Royal Free Hospital (RFH-NPT) has been found to exhibit a correlation with clinical deterioration, as well as the severity of disease, including the Child–Pugh score and the model for end-stage liver disease (MELD) score. Additionally, clinical complications such as ascites, hepatorenal syndrome, and episodes of HE have also been associated with the RFH-NPT score. It has been observed that the improvement in the RFH-NPT score is linked with enhanced survival and has been identified as an independent predictor of clinical deterioration as well as transplant-free survival [61]. The Liver Disease Undernutrition Screening Tool (LDUST) is a second liver-specific nutrition screening tool. The LDUST is based on six patient-directed questions regarding nutrient intake, weight loss, subcutaneous fat loss, muscle mass loss, fluid accumulation, and decline in functional status. However, it relies almost completely on the patient’s subjective judgment and has low negative predictive value. As with RFH-NPT, it needs further validation [62]. After conducting a cirrhosis screening, it is crucial to perform a thorough nutrition assessment in high-risk patients to confirm their nutritional status, determine their specific nutrition needs, and identify any factors that can be adjusted for optimal nutrition support. In the case of negative screening results, it is advisable to repeat the evaluation annually at a minimum [2].

During the screening, patients with cirrhosis who are at risk of malnutrition should undergo a comprehensive nutritional evaluation to approve malnutrition and characterise their nutritional status. A registered dietician or nutritionist should ideally perform it. [63]. As suggested, any cirrhotic patient should undergo a nutritional assessment every 1–6 months in the outpatient setting; for inpatients, the assessment should be conducted at admission and periodically throughout the hospital stay [60]. The elements of a detailed nutritional assessment include the evaluation of a detailed dietary intake assessment, global assessment tools, and muscle mass evaluation [2]. Dietary intake can be assessed using 24-h dietary recall, which is simple to use and does not require a high level of literacy, or by repeated 24-h dietary recalls [64].

Another option is a 3-day food diary, which requires patients’ cooperation and the following of standardised instructions. The last option should be the preferred method because it relies the least on patient recall [60]. The technique of subjective global assessment (SGA) uses data obtained during clinical evaluation to determine nutritional status without using objective measurements. It consists of five anamnestic parameters (weight loss, dietary changes, functional capacity, gastrointestinal symptoms, and metabolic demand associated with the underlying disease) and three pieces of physical examination data (oedema/ascites, loss of subcutaneous fat, and muscle wasting) [65]. Patients are assigned a rating of A (well-nourished), B (moderately malnourished), or C (severely malnourished) using the SGA method. While SGA is a straightforward and reproducible method [66], it tends to underestimate sarcopenia prevalence and has low agreement with other nutritional assessment methods. Nonetheless, it has been shown to correlate with post-surgery outcomes in patients without liver cirrhosis [67].

The Royal Free Hospital-global assessment (RFH-GA) [65] is a highly reproducible measure that aligns with other methods of body composition analysis. It also has the ability to predict post-transplant complications and survival rates [68]. Patients are stratified into one of three categories based on their dry weight-based Body Mass Index (BMI) and Mid-arm Muscle Circumference (MAMC): adequately nourished, moderately (or suspected to be) malnourished, or severely malnourished. However, it should be noted that the limitations of this tool include the significant amount of time required to administer it and the need for trained personnel to achieve accurate results. Therefore, further external validation is required before this valuable tool can be widely accepted.

### 4.3. Body Compositions and Muscle Assessments

Patients who are at high risk of malnutrition and obese patients (BMI >30 kg/m^2^) should undergo an anthropometric evaluation [2]. Malnutrition and sarcopenia are independent predictors of poor outcomes in patients with liver cirrhosis and those undergoing LT [69]. In addition, sarcopenic obesity and myosteatosis are independently associated with long-term mortality in liver cirrhosis, and frailty in cirrhosis is associated with increased mortality [45,70]. Sarcopenia can be assessed by radiologic methods (DXA, CT) to detect muscle mass loss or by muscle function tests [47]. CT images of the skeletal muscle area taken at the level of the 3rd or 4th lumbar vertebra can be measured and normalised for stature. Skeletal muscle mass on CTs has been associated with increased mortality [71]. CT-assessed muscle mass measurement in liver transplant patients showed an association between low muscle mass and mortality independent of the Model for End-Stage Liver Disease Score, as shown in a recent meta-analysis [14]. To avoid costly and invasive radiologic imaging, ultrasound-based protocols for evaluating muscle mass have been proposed [72] and need further validation. Ultrasound elastography using bi-dimensional shear wave elastography (2D-SWE) is a novel technology utilised for the measurement of tissue stiffness [73]. Pilot studies have demonstrated that skeletal muscle stiffness measurement is feasible and decreases with ageing, which correlates with muscle weakness [74]. According to a recent study, the feasibility and reproducibility of measuring rectus femoris muscle stiffness (RFMS) using 2D-SWE has been established in cirrhosis patients. It has been observed that RFMS is highly variable within each LFI class and exhibits poor correlation with muscle diameter. This suggests that it possesses complementary properties and could serve as a potentially simple, quantitative, and non-invasive test for stratifying muscle quality, thus warranting further investigation [75]. Dual-energy X-ray absorptiometry (DEXA) of the whole body is a method utilized to quantify bone mineral density, as well as fat and fat-free mass in patients with chronic liver disease. This technique is cost-effective and poses fewer risks of radiation exposure to patients, which makes it an ideal option for repeat testing [76]. However, it is less precise than a CT scan and not recommended for instances of fluid retention, which can lead to an underestimation of sarcopenia. In such cases, lean arm mass can serve as a more accurate alternative for determining mortality rates [77].

Body composition assessment involves the use of Bioelectrical Impedance Analysis (BIA), which measures the conductivity of hydrated tissues and estimates lean body mass through an equation. A recent study conducted [78] suggests that BIA is associated with mortality in compensated cirrhosis. However, the Phase Angle BIA method still requires validation in comparison to cross-sectional imaging, which is considered the standard gold method. Anthropometric measures, such as Mid-Arm Muscular Circumference (MAMC) and Triceps Skinfold Thickness (TSF), have also been utilized to measure lean muscle mass and body fat. These measures have been shown to predict malnourishment and lower survival rates in cirrhosis patients [79]. However, the accuracy of these measures can be affected by fluid retention. Recent studies [38,80] indicate that MAMC, TSF, and BMI were not independently associated with survival in patients with decompensated cirrhosis. The latest guidelines on sarcopenia by EWGSOP [81] propose a new three-step definition.

The recently updated guidelines emphasize the significance of functionally evaluating and quantifying muscle mass. In the event of reduced muscle strength, the possibility of sarcopenia should be considered. To aid in this, two clinical tools have been proposed: the hand grip strength (HGS) test and the chair stand test (CST). The HGS test involves taking the average value (in kilograms) of three consecutive measurements of the dominant arm using a dynamometer. The CST measures how many times a patient can stand up and sit down within 30 s. Both tests focus on identifying depletion of lean mass and low muscle strength. Although HGS is an independent prognosticator of mortality [82,83], it has a weak correlation with muscle mass and quality as assessed through cross-sectional imaging [84]. The recommended cut-offs for these tests remain unclear, as neither the European Association for the Study of the Liver (EASL) nor the ESPEN provided any in 2018 [2]. The second step in diagnosing sarcopenia involves the estimation of muscle quantity. Utilizing specific software, the skeletal muscle area (SMA) in cm^2^ can be quantified via CT scan imaging. The SMA is subsequently normalized to the squared height to calculate the skeletal muscle index (SMI) in cm^2^/m^2^. This technique can be easily performed as the abdomen is the object of diagnostic imaging in cirrhosis during routine follow-up, and it discriminates ascites from soft tissues. The lumbar skeletal muscle area is relatively well correlated with the whole-body muscle mass, particularly at the third lumbar vertebra (L3). It has been demonstrated that the software used or the injection of intravenous contrast has no effect [85]. In patients awaiting LT, SMI has been linked to mortality in cirrhosis [86]. The optimal cut-offs were determined to be 50 cm^2^/m^2^ for men and 39 cm^2^/m^2^ for women [87]. The best cut-offs were 50 cm^2^/m^2^ for men and 39 cm^2^/m^2^ for women. The choice of these values has been proposed by the EASL [2]. Some authors have proposed measuring the psoas major’s dimensions and surface area. It has a great advantage because it does not require special software. Durand et al. [88] also showed a significant association between transversal psoas muscle thickness normalised to height and mortality on the LT waiting list, independently of MELD. However, some physicians pointed out the weaknesses of this tool, including poor correlation with the total lumbar muscle area and a high measurement error [89]. Lastly, the severity of sarcopenia can also be estimated with physical performance. Gait speed, the short physical performance battery (SPPB), the timed-up-and-go test (TUG) or the 400 m walk are available in geriatrics. Physical performances (gait speed and SPPB) in patients waiting for LT have been associated with overall survival [90]. Currently, there are no standardised criteria for diagnosing cirrhosis frailty. The Liver Frailty Index (FI) model, developed by Rockwood et al., utilizes handgrip strength (HGS), the short physical performance battery (SPPB), and gender to establish an index that quantifies deficits [91]. This model provides a uniform approach to assess frailty, which can be applied to different patient cohorts [92]. Incorporating the FI in research pertaining to solid-organ transplant populations would facilitate the comparison of prevalence rates and adverse consequences across diverse groups [93]. It was demonstrated that FIs correlated with mortality in the LT context [94]; compared with non-frail patients, frail LT recipients had a higher risk of post-LT death and greater post-LT healthcare utilisation, although overall post-LT survival was acceptable [95]. Moreover, measures of frailty could be useful in identifying patients at higher risk for short-term rehospitalisation [96]. The presence of frailty in individuals with both compensated and decompensated cirrhosis is a significant predictor of disease progression, mortality, and unplanned hospitalization. Further exploration is required to determine the efficacy of interventions to prevent or reverse frailty in order to delay the advancement of cirrhosis [97].

## 5. Malnutrition and Sarcopenia in Orthotopic Liver Transplantation (LT) Candidates

The effect of malnutrition and sarcopenia on the development of cirrhosis complications is well-established, but their effect on the prognosis of liver transplant candidates and their role in liver transplant allocation is controversial [98].

The current liver transplant allocation system is based upon the Model for End-Stage Liver Disease (MELD) score used to predict short-term mortality in people with cirrhosis, which is based on serum bilirubin, the international normalised ratio of prothrombin time, and serum creatinine [99]. However, the MELD score appears inadequate in prioritising critically ill patients. In fact, in patients with ACLF, the CLIF-C organ failure score, which includes age, serum sodium, white blood cell count, creatinine, and INR, is a more accurate predictor of prognosis than MELD [100].

Moreover, the MELD-Na score, which incorporates serum sodium into the original score, has a more precise prognostic ability than the MELD score alone due to the fact that it accounts for hyponatremia, which is often associated with ascites, hepatorenal syndrome, and liver-related mortality [10]. Neither model, including the new MELD (5vMELD), which incorporates serum albumin and sodium (Na), or the MELD 3.0, the evolution of 5vMELD that takes gender differences into account [101,102], considers overall frailty and both lack an objective parameter that reflects the physical and nutritional status of the patients.

As proof of the significance of muscle mass as a prognostic factor, some new scoring systems, such as the MELD-Psoas and MELD-Sarcopenia scores, have been developed. The MELD Psoas score includes the L3-level psoas area normalised for stature, called the Psoas Muscle index (PMI), whereas the MELD Sarcopenia score takes into account the L3-level muscle area normalised for stature (SMI). SMI and PMI are calculated semi-automatically on transverse CT images using dedicated software [103]. Montano Loza et al. [104] demonstrated that adding sarcopenia to MELD (MELD-sarcopenia) facilitates the prediction of death in cirrhotic patients. When MELD was modified to include sarcopenia, it was most beneficial for individuals with low MELD scores, who are typically considered to have a low mortality risk. When present, sarcopenia adds 10 points to the MELD score, demonstrating its relevance [104]. Giving some priority to patients with sarcopenia before they develop extreme muscle depletion may reduce waiting list mortality in a subset of patients with cirrhosis without impairing LT survival [103]. Lai Jennifer C. et al. [105] highlighted the significance of considering the patient’s overall condition in the pre-LT evaluation. They considered the frailty index (including grip strength, chair stands, and balance) and demonstrated that in comparison to MELD-Na alone, MELD-Na plus the Frailty Index correctly re-classified 16% of deaths/delisting (*p* = 0.005) and 3% of non-deaths/delistings (*p* = 0.17) for a total NRI of 19% (*p* = 0.001) [105]. In addition, it is important to consider the role of sarcopenia in LT waiting list patients. In liver transplant candidates with cirrhosis, sarcopenia is associated with mortality on the waiting list, particularly in patients with lower MELD scores [104,106]. A recent study has developed a model called “Sarco-Model_2_” to predict dropout risk and determine an appropriate post-LT futility threshold in cirrhotic liver transplant candidates. This model combines sarcopenia and MELD-Na and has shown good diagnostic ability in predicting the risk of dropping out after three months in sarcopenic patients with a MELD-Na <20. However, for sarcopenic patients with a MELD-Na of 35–40, the model suggests a high graft loss risk and recommends “futile” transplantation [107]. The assessment of the muscular area at L3 with a CT scan (Skeletal Muscle Index, SMI) is the gold standard for the diagnosis of sarcopenia [14], but its use in clinical practice is limited. Mauro E. et al. validated the Sarcopenia HIBA score based on four variables: sex, BMI, Child–Pugh score, and creatinine/cystatin C ratio, and they demonstrated that it is an easy-to-use, objective, and reliable diagnostic and predictive tool that can be used to improve prognostic evaluation and to identify patients with a higher risk of death while awaiting LT [108]. Additionally, sarcopenia is associated with life-threatening complications, such as acute on chronic liver failure (ACLF), that can occur during LT waiting and result in death. Similarly, cystatin C has a similar predictive function [109].

Malnutrition is associated with a greater risk of postoperative complications and mortality rates in patients with chronic disease, and this correlation is also suggested in cirrhotic patients undergoing LT [110].

The literature confirms that sarcopenia is associated with higher post-LT mortality [68,111,112,113,114]. Although most studies consider psoas muscle mass index (PMI) as the indicator of sarcopenia, the correlation with survival is confirmed using both quantity and quality measurements. For example, Hamaguchi et al. considered intramuscular adipose tissue content (IMAC) and PMI together, and they found that high IMAC (OR = 3898) and low PMI (OR = 3365) were independent risk factors for death after LDLT [115].

This evidence contrasts with a few studies that did not find a correlation between muscle depletion and survival [116,117]. Furthermore, some trials highlighted a different impact of sarcopenia in post-OLT mortality between men and women [98,118,119].

Moreover, research has demonstrated that sarcopenia and malnutrition are also associated with different post-LT complications, such as a higher risk of post-LT infections [120], longer mechanical ventilation duration [112], longer lengths of stay in the ICU and the hospital [68], and mortality [118].

In conclusion, further studies are necessary to identify objective criteria for assessing global health status to avoid futile liver transplants. Measures of malnutrition and sarcopenia may be particularly interesting because they are objective and reproducible, and several studies have demonstrated their correlation with post-LT outcomes. Multicenter and prospective studies may be useful to evaluate cut-offs specific to age and sex that are able to stratify post-LT risk.

## 6. Nutritional Management Strategies before LT Patients

Based on these previous considerations, malnutrition, sarcopenia, and obesity should not only be investigated and assessed in patients with liver cirrhosis but also specifically managed in those awaiting LT.

As a matter of fact, protein malnutrition, sarcopenia, and obesity in patients listed for LT are associated with increased mortality and morbidity [68,112,121,122,123,124,125,126].

At least 60% of cirrhotic patients awaiting LT are malnourished, mainly because of insufficient food intake [127,128]. Indeed, Ferreira et al. demonstrated that a negative energy balance (obtained by subtracting total caloric intake from total energy expenditure), mostly due to inadequate food intake, was present in 78.1% of cirrhotic patients awaiting LT [129]. In particular, patients consuming a low-protein diet have a higher mortality risk waitlisted for LT since protein intake below 0.8 g/kg/d has been identified as an independent predictor of transplant waiting list mortality [130].

Despite these findings, as far as we know, to date, except for a single study recruiting patients undergoing living donor liver transplantation (LDLT) [131], none of the studies evaluating nutritional supplementations have shown significant benefits in post-LT “hard” outcomes.

Le Cornu et al. [132] performed a prospective randomised study in patients awaiting LT, comparing supplementation of 750 kcal and 20 g of protein per day associated with nutritional counselling alone. However, this strategy, even though it demonstrated an improvement in mean arm circumference, arm muscle circumference, and grip strength, had no impact on mortality in the post-LT period [132].

Plank et al. [133] conducted a pilot study evaluating pre- and post-LT administration of an immunonutrition diet (IMD). Patients received oral nutritional supplements (ONS) containing immune nutrients such as arginine, omega-3 fatty acids, and ribonucleic acid (Oral Impact^®^) until LT, for a median of 54 days. After surgery, as soon as tolerated, patients were administered enteral Impact^®^ until oral intake provided more than 50% of energy requirements. Supplementation with Oral Impact^®^ was started for at least 5 five days after surgery. This protocol improved protein stores and resulted in a lower rate of post-operative infections [133]. This result was unfortunately not confirmed by a subsequent randomised, double-blind trial, in which perioperative IMD did not improve preoperative nutritional status, assessed as a change in total body protein, or postoperative outcomes of patients undergoing LT [134].

The latter study was part of a meta-analysis including seven randomised controlled trials involving more than 500 patients and evaluating the effects of perioperative IMD on clinical outcomes in patients undergoing LT. The authors demonstrated a significant reduction in infectious complication rate and postoperative length of hospital stay but failed to show differences in mortality [135].

On the other side, two retrospective studies evidenced for the first time a possible correlation between oral branched-chain amino acid (BCAA) administration and infectious complications in LDLT. The length of administration and dosage differed in the two studies. Shirabe et al. [136] analysed 100 consecutive patients undergoing LDLT. Two weeks before LDLT, 37 patients received a BCAA-enriched nutrient mixture (Aminoleban EN^®^, 100 g/d), and 28 received BCAA nutrients (Livact^®^, 12.45 g/d). Thirty-five received no nutritional therapy. A reduction of post-LT bacteremia was demonstrated in patients supplemented with a BCAA-enriched nutrient mixture [136]. Kaido et al. [137] evaluated 236 LDLT recipients. For over one month before transplantation, 86 patients were administered three packets of Livact^®^ per day, while 43 patients took 1 to 3 packets of Aminoleban EN^®^ per day (50–150 g/day). One hundred and seven received no supplementation therapy. The study highlighted an amelioration of post-LT sepsis in LDLT patients undergoing a preoperative oral BCAA supplementation without any significant difference between Aminoleban EN^®^ and Livact^®^ [137].

Moreover, Grat et al. [138] conducted a prospective randomised study administrating a daily multi-strain probiotic (*Lactococcus lactis*, *Lactobacillus casei*, *Lactobacillus acidophilus*, and *Bifidobacterium bifidum*) to the interventional arm from listing to LT. This approach seemed to significantly reduce the rate of 30 day and 90 day postoperative infections but did not decrease postoperative mortality [136]. A similar result on infectious complications was obtained by Eguchi et al. with a perioperative synbiotics therapy (*Bifidobacterium breve*, *L. casei*, GalactoOligoSaccharides) in patients undergoing LDLT, compared to the standard of care [139].

Kaido et al. [131] reported a significant improvement in 5-year overall survival in patients with sarcopenia undergoing LDLT who received a combined perioperative nutritional therapy. Before LT, for two weeks, patients assumed a BCAA-based late evening snack (Aminoleban EN^®^ or Livact^®^), synbiotics (GFO; glutamine, dietary fibre, oligosaccharide) three times a day, a lactic fermented beverage (Yakult 400^®^, containing *L. casei*) once a day, and a zinc supplement (Promac D^®^, in case of zinc deficiency). Twenty-four hours after surgery, post-operative early enteral nutrition through jejunostomy was started with a gradual increase in daily caloric intake. The enteral nutrition regimen was based on an immune-modulating diet (IMD) enriched with hydrolysed whey peptide (MEIN), a protein complex derived from milk. The enteral feeding median duration was 21 days (12–92 days), and it was continued until the patient could tolerate adequate oral intake [131].

Due to the large heterogeneity of the reported studies and the small sample size, more evidence may be needed to support a statement. In the studies, there was no uniform method for assessing malnutrition or sarcopenia, and some studies included both malnourished and non-malnourished patients, resulting in a uniformity bias in both patient enrolment and outcome analysis. In addition, the nutritional protocols varied in terms of the type of nutritional supplementation (e.g., oral versus enteral) and duration (pre-LT vs perioperative nutritional supplementation). Lastly, the majority of studies analysing nutritional management before LT included only LDLT candidates (ed., LDLT is planned surgery); therefore, patients scheduled for LDLT were suitable for enrolment in clinical trials, as adequate preoperative nutritional intervention can be completed within a specific time frame, in contrast to deceased donor liver transplant (DDLT). To better understand the role of pre-LT nutritional strategies in modifying clinical outcomes in patients awaiting LT, larger randomised controlled trials with precise patient selection and nutritional assessment are required.

To prevent the onset of malnutrition, sarcopenia, and frailty, however, the nutritional status of patients with cirrhosis, especially those awaiting LT, must be improved in every way to prevent malnutrition. Given the significance of correctly achieving nutritional goals, this is best accomplished by a team of expert clinical nutritionists [140].

As no randomized-controlled trials showing that perioperative nutritional strategies improve clinical outcomes in patients awaiting LT have been published, no specific recommendations can be given. However, in light of the evidence collected so far, improving nutritional status in every aspect to prevent the onset of malnutrition, sarcopenia, and frailty needs to be a primary goal in managing patients with cirrhosis, especially those awaiting LT. Recent guideline of the ESPEN suggest an energy intake of 30–35 kcal/kg/day and a high protein intake (1.2–1.5 g/kg/day of protein), preferably through the oral route [122]. A total amount of 1.5 g/kg/day of protein may be preferred in sarcopenic patients [122]. Given the importance of achieving nutritional goals correctly, it becomes apparent that this is best carried out with a team of expert clinical nutritionists [140].

In the setting of obese (non-hospitalized, clinically stable) cirrhotic patients, however, according to the American Association for the Study of Liver Disease (AASLD), daily caloric targets should be stratified by BMI: 25–30 kcal/kg of ideal body weight in patients with a BMI of 30–40 kg/m^2^ and 20–25 kcal/kg of ideal body weight in those with a BMI ≥ 40 kg/m^2^ [33]. While weight loss should be obtained by reducing carbohydrate and fat content, a high protein intake (1.2–1.5 g/kg of ideal body weight) must be maintained [141]. Small and frequent meals (every 3–4 h) during the day and a late evening snack have become key points to preventing accelerated starvation. They improve nitrogen balance, reduce skeletal muscle proteolysis, and increase total body protein [127,142,143].

Although there is no conclusive evidence that physical activity leads to better outcomes while on the LT waitlist, it is critical for patients with cirrhosis to maintain muscle mass and improve muscle function with regular physical activity. Formal guidelines for physical activity in cirrhosis still need to be improved, along with the need for large and well-designed clinical trials. The expert consensus is that the optimal activity-based intervention should include aerobic and resistance training [144]. However, since deconditioning and fatigue represent significant obstacles in patients with advanced chronic liver disease, exercise intervention should be patient-tailored [144].

Debette-Gretien et al. [145], for the first time, conducted a prospective study assessing the impact of a 12-week personalised adapted physical activity protocol in a small cohort of patients awaiting LT. They showed, on the one side, the safety of the physical activity and, on the other side, an amelioration of aerobic capacity and muscle strength in patients awaiting LT [145]. Similarly, Wallen et al. [146] demonstrated the safety and feasibility of an 8-week exercise training (both aerobic and circuit-based resistance exercises) in a small pilot randomised controlled trial with patients awaiting LT. However, no significant changes in intraoperative, perioperative, or postoperative outcomes were highlighted [146]. This study is part of a systematic review and meta-analysis conducted by Jetten et al. [144], who analysed eight studies involving over 1900 patients. The authors evaluated the physical effects, safety, and practicability of preoperative exercise programs in both compensated and decompensated LT candidates. The examined exercise programs varied in duration, exercise mode (supervised versus unsupervised), frequency, and type of exercise (aerobic, strength, and walking). Significant improvements in peak VO2, 6-min walking distance, hand grip strength, liver fragility index, and quality of life were observed regardless of the training program [138].

Additional evidence is required to improve physical activity programs for patients with chronic advanced liver disease and to support the safety and efficacy of exercise in decompensated patients. However, personalised physical activity programs appear feasible and promising even in patients awaiting LT.

## 7. Nutritional Management Strategies after LT

It is originally expected that LT would modify the nutritional and body composition abnormalities that characterise the clinical condition of patients with chronic liver disease [147]. Several factors may influence these aspects after LT, the most significant of which is allograft function; in the event of primary nonfunction or graft rejection, many of the pre-LT nutritional alterations will persist, but even in a well-functioning graft, some nutritional disturbances and body composition alterations will not fully normalise over the long term despite the recovery of liver function [147]. The liver–gut–brain axis is linked to this concept and acts as a nutritional factor after LT [148]; after LT, the liver becomes isolated from the nervous autonomic regulatory control, suggesting that this isolation could influence nutrient absorption, glucose and lipid homeostasis, appetite signalling, and eating behaviour [149], thereby contributing to shaping body composition and altering weight [147]. In fact, after LT surgery, energy and protein requirements remain elevated for weeks [150]; subsequently, in the phase immediately following transplant, protein catabolism is frequently elevated alongside impaired protein synthesis [151,152], resulting in the persistence of sarcopenia for up to a year or longer [153,154,155,156]. The increase in resting energy expenditure (REE) can persist for a long time [157,158], despite the occurrence of overweight and obesity during long-term follow-up [159].

### 7.1. Nutritional Support Immediately after LT

All transplant patients spend a few days in the surgical intensive care unit (SICU) to be adequately supported and monitored [160]. During this immediate recovery, nutritional support should always be taken care of to re-establish graft function and shorten the overall recovery time, which has to deal with the stress of critical illnesses and multiple treatments (mechanical ventilation, hemodiafiltration, use of corticosteroids) [147]. Liver transplant recipients experience improved metabolisms within four weeks, as seen in changes to non-protein respiratory quotients, serum non-esterified fatty acids, and nitrogen balance [147,161]. The protein catabolism is markedly increased in the immediate phase after LT. The patients should receive about 1.5–2.0 g/kg of protein [38,143]; this protein intake recommendation is particularly relevant in the presence of fistulas or surgical drainage [161]. Therefore, adequate protein and energy intake provisions should be ensured during the acute post-LT phase to avoid protein breakdown, metabolic syndrome, and sarcopenia [161,162]. Hypermetabolism has been shown to predict transplant-free survival independently of MELD and Child–Pugh scores and tends to continue for at least one year post-LT [163].

Post LT, patients should start normal food oral intake or enteral feeding within 12–24 h, depending on their tolerability. Parenteral nutrition should only be contemplated as an ultimate measure to fulfil everyday calorie and macronutrient demands in the presence of malnutrition or in the absence of oral feeding feasibility [164,165]. The total daily energy intake until the third postoperative day (POD) is 10–15 kcal/kg and gradually increases to 25–35 kcal/kg [2,161,165]. Lipids are the preferred energy sources in the early postoperative hours, as glucose is not processed well. Indeed, a reduction in glucose utilisation by the graft was observed in the first six hours of engraftment due to impaired mitochondrial respiration and inactivity of the tricarboxylic acid cycle [166,167]. During this time, energy is mainly generated by the oxidation of fatty acids [167,168]. After six hours, glucose administration could then be initiated [161,162], although it has been recommended in small doses and without insulin buffering to not suppress peripheral fat mobilisation, judged clinically by blood glucose levels, lactate, triglycerides, and arterial ketone bodies [167,168,169].

The resumption of enteral nutrition (EN) within 12 h of LT has been shown to reduce postoperative viral and bacterial infections [137,147,170] and produce lower nitrogen excretion as a marker of reduced protein catabolism [133,147]. Around POD five, patients can generally swallow food and tolerate adequate oral intake containing solid foods. EN should only be stopped once patients maintain adequate oral intake consistent with their nutritional needs [131,170,171]. EN formulas should be enriched with protein, semi-elemental formulas with small peptides, and medium-chain triglycerides if malabsorption is present [161,162]. Regarding nutritional supplements nowadays, there is no clear evidence of their benefit after LT [164]. Even after LT, the IMD continues to improve post-LT outcomes by a series of mechanisms; the benefits of an IMD added to an EN or PN are based on its ability to downregulate the production of inflammatory cytokines to modulate the eicosanoid synthesis and to improve post-LT immunosuppression [133,172]. Therefore, IMD could improve ischemia or reperfusion damage of transplanted organs [10]. Conversely, arginine can improve immune function and nitrogen balance and stimulate wound healing [133]. An IMD enriched with hydrolysed whey peptide (HWP) has decreased post-LT bacteremia, infections, hyperglycemia, and post-LT mortality compared to the conventional IMD [170].

### 7.2. Dietary Recommendations in Patients on Immunosuppressive Therapy

The liver transplant recipient receives lifelong immunosuppressive therapy to minimise the risk of transplant rejection [173]. Therefore, among immunosuppressant drugs, special attention should be paid to corticosteroids due to their property of increasing hunger, fat deposition, proteolysis, impaired protein synthesis, and reduction of fat oxidation [174]; calcineurin inhibitor drugs (CNI) such as cyclosporine have also been found to be an independent predictor of post-LT weight gain [147,174], while tacrolimus has been reported to increase basal energy expenditure (BEE) [175].

CNIs may contribute to impaired muscle growth and regeneration by inhibiting calcineurin, which affects skeletal muscle differentiation, hypertrophy, proteolysis, and fibre type determination [174,175]. The mammalian target of rapamycin (mTOR) inhibitors, sirolimus and everolimus, can reduce muscle mass [175]. After LT, many patients may have concomitantly elevated potassium levels, usually caused by the nephrotoxicity of immunosuppressive therapy [176]. Therefore, in the early post-transplant period, it should be important to monitor dietary sources of potassium (especially fruits and vegetables such as leafy greens, beans, nuts, dairy products, and starchy vegetables such as winter squash) and recommend the use of dietary techniques capable of reducing its nutrient content [143]. However, these suggestions are intended to be temporary.

Hypomagnesemia is also a consequence of immunosuppression (by affecting magnesium transport along the distal convoluted tubule, causing renal magnesium wasting [177]), and therefore the intake of magnesium-rich food sources, such as dark cocoa, whole grains, nuts, legumes, fruits, and vegetables, should be encouraged [175].

Patients who receive immunosuppressive therapy are more susceptible to infections, and therefore rigorous food safety advice should be adopted to prevent food-related infections [159]. After LT, the patients are 15–20% more exposed to foodborne illnesses than the general population, and consequently, proper handling of fruits and vegetables is mandatory [147,175]. Generally, it is advisable to choose pasteurised foods, wash hands frequently, avoid raw meat or canned food, and maintain an adequate food storage temperature [137,159]. In addition, several foods containing compounds capable of modulating cytochrome activity, such as grapefruit, turmeric, pomelo, ginger, pomegranates, Seville oranges, black pepper, cranberry juice, black tea, beer, cruciferous vegetables, kava, the root of liquorice, wine, and olive oil, can act on the pharmacokinetics/pharmacodynamics of immunosuppressive drugs, leading to altered levels of immunosuppressants in the blood [178].

### 7.3. Diet and Calorie Intake in LT Patients

Most published studies have reported a significant increase in caloric intake after LT. This is especially evident in those patients who followed long-term and severe dietary restrictions or those who suffered from relevant gastrointestinal symptoms or anorexia before LT [147,157,179]. From nine months up to 2 years after LT, patients’ weights exceeded pre-illness values on average by 7.5 kg or 8% of body weight [180]. The observed reduction in BEE and increase in energy intake after LT results in a positive energy balance that contributes to weight gain and leads to a high prevalence of overweight and obesity in patients after a long time after LT (9 months after hospital discharge, 87% of patients; rate of 43% of transplanted after 18 months LT) [147,180].

Several authors have investigated the calorie intake trend: in the first year after LT, calorie intake increased, according to the majority of studies, from a minimum of 1542 ± 124 kcal/day to a maximum of 2227 ± 141 kcal/day due to higher protein (from 60.3 g/day to 83.4 g/day) and carbohydrate (from 199 g/day to 261.8 g/day) consumption and approximately doubled fat intake (from 62.3 g/day to 101.6 g/day) compared to pre-LT patients [179,181,182]. In 84 LT recipients, the increase in caloric intake 12 months after LT was associated with decreased carbohydrate intake and increased fat consumption, as reported by Lunati et al. [183]. McCoy et al. [184] evaluated dietary intake before LT and 6 and 12 months after it in 17 consecutive LT recipients, but they did not observe significant changes in total energy, protein, or lipid intake after LT, except for carbohydrates, where the intake was lower compared to pre-LT. Nonetheless, studies showed conflicting results in long-term LT follow-up. A prospective study of 117 patients evaluated four years after LT found a significant increase in caloric intake (1920.9 ± 633.1 kcal versus 2016.7 ± 666.1 kcal) and macronutrient intake [185]. Conversely, in a study by Ribeiro et al. [186], 42 patients who were followed up for more than 6.5 years after LT showed a lower energy intake of 1620.9 ± 457.0 kcal, despite only 36.8% of those patients showing correct caloric intake according to BEE.

Merli et al. [181] hypothesised that energy intake might differ depending on previous LT nutritional status. They studied 25 post-LT patients and discovered that dietary intake increased only in previously malnourished patients (from 27 kcal/kg/day before LT to 32 kcal/kg/day 12 months later LT). Twelve months after LT, a decrease in carbohydrate intake and an increase in lipid intake were observed. Protein consumption increased three months after LT (from 0.88 to 1.34 g/kg/day) and remained stable at six and twelve months after LT. Conversely, in patients without malnutrition before LT, the macronutrient intake was comparable before and after LT, and the BEE, which was negative before the liver transplant, began to improve around the third month after LT.

### 7.4. Long-Term Nutritional Support after LT

Patients can lose approximately 9 kg during chronic liver disease, so it is essential to regain optimal nutritional status [187]. By six months after LT patients have regained most of their lost body weight, but only after one year do they regain all of it and even exceed it in the following years, resulting in overweight and obesity [147,162,188]. Unfortunately, despite the weight gain, sarcopenia does not improve after transplantation [2,137,180]. Weight gain depends on fat mass, whereas muscle mass recovery is subtle and non-significant by the end of the first year [180,189]. On the one hand, an increase in BMI after LT is an independent positive predictor of improved patient and graft survival after LT [156,190]. A study of 2968 liver transplant recipients who were not overweight prior to their transplant found that 54% of patients experienced an increase in BMI within two years after the transplant. Patients who experienced an increase in BMI had a significantly higher 5-year survival rate (90.1%) and better engraftment (89.4%) when compared to those who either maintained or lost BMI (82.0% and 77.2%, respectively) [190].

In contrast, metabolic syndrome, hyperlipidemia, and obesity are prevalent in patients six months after LT, particularly if immobilisation occurs. This is linked to an increased risk of major cardiovascular events, diabetes, hypertension, cancer, and fibrosis progression. These conditions contribute to long-term morbidity and mortality [137,187,188]. Weight gain after LT is characterised by an early and inappropriate gain in fat mass, with a slow and incomplete restoration of body cell mass [182,191]. This may favour sarcopenic obesity and not necessarily an improvement in nutritional health [192].

It is recommended to educate patients about consuming a diet that fosters a favourable body composition by limiting fat intake and consuming adequate amounts of lean protein sources to encourage muscle development. To prevent water retention induced by steroid therapy, a “no added salt” diet with a daily maximum of 3 g of sodium is advised. Furthermore, it is necessary to ensure that calorie intake is adequate to avoid protein utilization as an energy source, yet not excessive beyond the body’s energy requirements [193]. Dietitians should reassess nutritional status to optimise the patient’s diet during the transition from the acute to chronic post-LT phase and ensure patient compliance [147]. This regular follow-up is really important due to malnutrition being a condition characterising not only the pre-LT status (up to 84.8%) but also eventually the post-LT one (at three months, 46.9% of patients; at six months, 25.8%; at 12 months, 10.7%) [194].

### 7.5. Eating Behaviour and Psychological Factors

Data regarding eating disorders in LT patients and the impact of psychological factors on eating behaviour are limited. Psychological status can heavily influence different stages of LT [195]. Patients on the LT waitlist are characterised by stress and anxiety [196]. Alterations in eating behaviours could result from dietary restrictions before and after LT, such as unnecessary protein restriction, sodium restriction, and diets low in potassium and carbohydrates [60,130,197]. Tighter food control is known to lead to eating disorders such as anorexia nervosa (AN), bulimia nervosa (BN) [197], and binge eating disorder (BED) [198]. Furthermore, negative feelings or depressive syndrome [199] after LT can lead to and be mediated by dietary restrictions [200]. In particular, emotional eating is known to influence BMI [201] and is linked to depression [202,203].

Eating behaviour, weight gain, and excess were evaluated in 301 recipients in a study that assessed eating behaviour in LT patients [204]. The obese patients scored significantly higher in eating behaviour tests than nonobese patients with uncontrolled eating, cognitive restraint, and emotional eating.

A study evaluating food addiction and abuse in 236 LT recipients found that 5.1% of LT patients were food-addicted, and 39.8% were food abusers [205]. On the other hand, some authors curiously did not find an association between weight gain and stress or depressive feelings, as 69% of the patients gaining weight did not show depressive feelings or eating disorders [184].

### 7.6. Physical Activity after Liver Transplantation

Immediately after LT, patients are instructed to mobilise. The elevated risk of cardiovascular disease, cancer, and osteoporosis in this patient population is reduced by dietary and physical activity modifications over the long term. Even though some transplant centres provide patients with dietary counselling, physical therapy, and activity advice, research indicates that many liver transplant recipients do not achieve the recommended levels of activity [143] or dietary intake, as evidenced by excessive weight gain and metabolic syndrome after transplant. Physical activity, self-care, and mobility were all associated with improved QOL after transplantation [206]. Independent of comorbidities, participation in group sports activities was associated with improved physical function and QOL up to 5 years after transplant [207]. There needs to be a consensus regarding physical activity goals for liver transplant recipients. Due to the importance of physical activity in reducing the risk of cardiovascular complications [182], it seems reasonable to recommend 150 min per week of moderate-to-vigorous activity and 15–20 min of resistance exercise training twice per week [182].

## 8. Role of Bariatric Surgery in LT Setting

Among liver disease etiology, NAFLD, progressing to NASH, is now one of the leading causes of end-stage liver disease requiring LT worldwide [208]. The last (2020) Annual Data Report of the Organ Procurement and Transplantation Network/Scientific Registry of Transplant Recipients (OPTN/SRTR) stated that candidates for LT with pre-obesity (BMI 25.0–29.9 kg/m^2^) composed 31.9% of the waiting list and candidates for LT with class I obesity (BMI 30.0–34.9 kg/m^2^) composed 22.3%, while candidates with class II and III obesity together (BMI ≥ 35 kg/m^2^) composed 17.0% of the waiting list, representing the only BMI class with a continuously increasing trend [209]. The mean BMI of patients undergoing LT reflects the increasing mean BMI of the general population [210]. Obesity is associated with some comorbidities, such as arterial hypertension and type 2 diabetes mellitus, impacting long-term outcomes in LT patients [211]. Moreover, even with appropriate perioperative and anaesthetic precautions, obese patients can undergo LT like people of normal weight; however, this condition is associated with impacting medical complications in the post-LT course [121,212,213]. Indeed, a recent meta-analysis suggested that WHO class III obesity leads to a significantly increased mortality risk after LT and that obesity generally exposes patients to a higher risk of postoperative complications compared to normal-weight patients [214].

As a result of these concerns, it is critical to understand how to manage this condition when individuals with liver disease and obesity may be candidates for LT. Currently, barring a profound lifestyle change, the most effective treatment for morbid obesity, and its comorbidities, is bariatric surgery (BS); this is also the case for cirrhotic patients. In this scenario, it is essential to perform a multidisciplinary evaluation, with a surgical and anaesthesia team able to manage liver-specific complications. A careful assessment of portal hypertension and liver decompensation is needed before accessing BS [215]. Indeed, although BS has been shown to be effective in obese cirrhotic patients, a detailed postoperative follow-up needs to be emphasised because rare liver impairment cases have been reported after bariatric surgery [216,217]. The mechanism underlining this phenomenon is unclear, and prognostic parameters are lacking.

The preferred bariatric technique for patients with cirrhosis is the sleeve gastrectomy (SG), which determines mechanical and hormonal weight loss. It consists of the removal of most of the body and the fundus of the stomach (typically from 60% to 75%) so that the lumen decreases in volume and, by resection of the gastric fundus, decreases ghrelin production and reduces hunger [218]. An endoscopic approach known as endoscopic sleeve gastroplasty (ESG) has proven effective in performing SG in cirrhotic patients [219]. What makes SG the best choice is the lack of interference with corticosteroid pharmacokinetics and easier access to the stomach in the event of gastric variceal bleeding [220]. Laparoscopic adjustable gastric banding (LAGB) and Roux-en-Y gastric bypass (RYGB) are otherwise effective types of weight loss surgery. LAGB consists of an adjustable band placed around the upper part of the stomach to create a very small gastric pouch, while RYGB consists of creating a small pouch from the stomach and connecting the newly created pouch directly to the small intestine [221]. Moreover, endoscopic bariatric therapies, mainly the intragastric balloon (IGB), have evolved as effective and safe tools for weight loss [222], even in cirrhotic patients awaiting LT. No worsening in varices and portal hypertensive gastropathy was observed after IGB, although adverse events can occur: a plot study showed symptomatic upper gastrointestinal bleeding in the setting of a Mallory Weiss tear in 12.5% of a patient [223]. Regardless of the BS techniques, it is crucial to emphasise that BS improves weight loss and hepatic steatosis [224]. Although it is currently evident that BS improves the outcomes of obese cirrhotic patients undergoing LT, the optimal timing of BS within the context of LT remains controversial [89,90].

When BS is performed before LT (pre-LT BS), the main benefit obtained is the resolution of obesity-related comorbidities. Concerning this point, BS may improve not only the accessibility to LT by lowering the BMI but also both short- and long-term LT outcomes [225]. However, this timing of BS implies increased costs, two different hospitalisations, increased patient discomfort, and delayed LT [226,227]. Moreover, pre-LT BS could determine worsening sarcopenia and malnourishment; thus, it is indicated only in patients with compensated cirrhosis. On the other hand, barring the complexity of the procedure, performing BS during the LT can minimise costs and patient discomfort by ensuring the resolution of obesity and related comorbidities after LT [228,229,230]. Nevertheless, obesity and concomitant diabetes represent additive risk factors and strong predictors of postsurgical respiratory and cardiovascular complications and infections [231]. Finally, when BS is performed after LT, the main advantage is the decrease in obesity-related comorbidities, while the main disadvantage is the increased risk of wound dehiscence and infection post-immunosuppression-LT [232,233]. SG is the leading technique because it does not affect the pharmacokinetics of immunosuppressive agents [234].

In conclusion, although obesity surely has negative long-term health consequences, the correlation between obesity and outcomes after major surgery, including LT, remains controversial. Performing BS on these patients, even with cirrhosis and portal hypertension, appears safe, with acceptable perioperative and long-term outcomes [235]; further studies are required to establish evidence-based and robust recommendations (Table 2). A multidisciplinary approach is crucial for improving outcomes for obese patients with cirrhosis undergoing LT. Additionally, the optimal timing of BS in the setting of LT has yet to be identified, and the literature has recorded only a few cases of BS in advanced liver disease. More studies with matched controls and longer follow-up are required to shed light on this matter.

## 9. Conclusions

**Figure 2:**
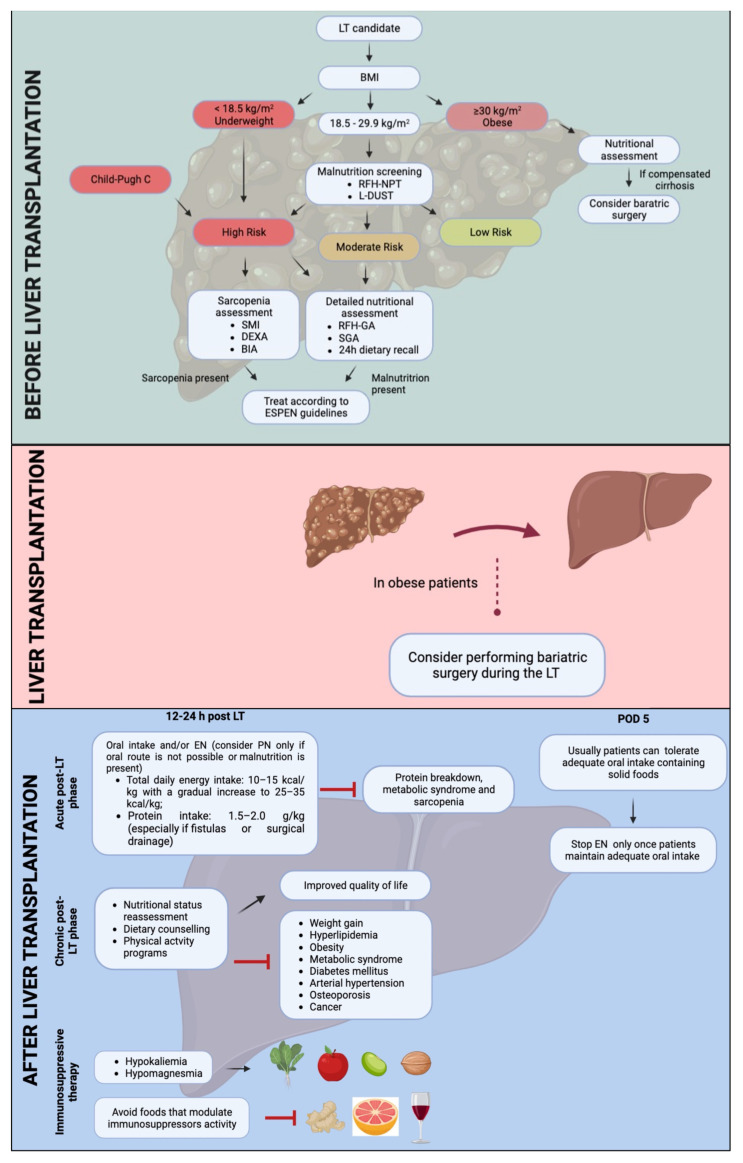
A pragmatic nutritional approach to the liver transplant patient in patients with cirrhosis.

In conclusion, malnutrition and sarcopenia are essential for patients with liver cirrhosis and their complications. All patients scheduled for a liver transplant must implement screening strategies for these conditions, even obese patients. In the transplant candidate and, ideally, in all chronically followed cirrhotic patients, multidisciplinary nutritional management is crucial for weight management and the correct and appropriate introduction of micronutrients/macronutrients (i.e., proteins and BCCA). Appropriate nutritional management of the pre-transplant patient can reduce the risk of recurrence and/or the development of post-LT metabolic disorders. Nutritional management must begin immediately after surgery and continue during the postoperative period. Therefore, any healthcare professionals working on LT patients must prioritise their patients’ nutritional needs to improve outcomes and significantly reduce the risk of complications. We confidently presented Figure 2, a pragmatic strategy that is thoroughly supported by the latest research findings.

## Figures and Tables

**Table 2:** A comparison of the benefits and risks of bariatric surgery techniques for patients with cirrhosis.

Techniques	Pro	Cons	Reference
*Sleeve gastrectomy (SG)*	Mechanical and hormonal weight loss. No interference with corticosteroid pharmacokinetics. Easier access in the event of gastric variceal bleeding. Maintains access to the biliary system.	Development of Gastroesophageal Reflux Disease (GERD).	Mittal et al., 2021 [220]
*Laparoscopic adjustable gastric banding (LAGB)*	Lower early complications and shorter operative time and length of stay.	Interference with corticosteroid pharmacokinetics. Not the most effective surgical procedure to reducing weight.	Tichansky et al., 2005 [221]
*Roux-en-Y gastric bypass (RYGB)*	Reduction of reflux gastritis and esophagitis. It improved glycemic control and high-density lipoprotein levels.	No access to the biliary system. Possible development of a stomal ulcer. Increased probability of cholelithiasis and Roux stasis syndrome. Interference with corticosteroid pharmacokinetics.	Tichansky et al., 2005 [221]
*Intragastric balloon (IGB)*	Non-invasive and rapid procedure. Rapid weight loss.	Upper gastrointestinal bleeding. Increase in liver fat fraction. Rapid weight loss. Not durable.	Watt et al., 2021 [223]
Timing	Pro	Cons	Reference
Before LT	Resolution of obesity-related comorbidities.	Increased costs. Two different hospitalisations. Increased patient discomfort and delayed LT. Worsening sarcopenia and malnourishment of the patient.	Diwan et al., 2018 [225]
During LT	Resolution of obesity-related comorbidities. Costs and patient discomfort are minimised.	The complexity of the procedure.	Tariciotti et al., 2016 [228]
After LT	Decrease in obesity-related comorbidities after LT.	Increased susceptibility to infections. Poor wound healing. Hostile abdominal environment after LT.	Lin et al., 2013 [232]

## Data Availability

Not applicable.

## Abbreviations

2D-SWE: Bi-Dimensional Shear Wave Elastography; AASLD: American Association for the Study of Liver Disease; ACLF: Acute on Chronic Liver Failure; ASPEN: American Society of Parenteral and Enteral Nutrition; BCAAs: Branched-Chain Amino Acids; BED: Binge Eating Disorder; BIA: Bioelectrical Impedance Analysis; BMI: body mass index; BN: Bulimia Nervosa; BS: Bariatric Surgery; CNI: Calcineurin Inhibitor drugs; CST: Chair Stand Test; CTP: Child–Turcotte–Pugh score; DEXA: Dual-Energy X-ray Absorptiometry; EASL: European Association for the Study of the Liver; EN: Enteral Nutrition; ESG: Endoscopic Sleeve Gastroplasty; ESPEN: European Society for Clinical Nutrition and Metabolism; GLIM: Global Leadership Initiative on Malnutrition; HGS: Hand Grip Strength; IGB: Intragastric Balloon; IMAC: IntraMuscular Adipose tissue Content; IMD: Immunonutrition Diet; LAGB: Laparoscopic Adjustable Gastric Banding; LDLT: Living Donor Liver Transplantation; LDUST: The Liver Disease Undernutrition Screening Tool; LT: Liver Transplant; MAMC: Mid-arm Muscle Circumference; MELD: Model for End-stage Liver Disease; MNA-SF: Mini Nutritional Assessment Short Form; MST: Malnutrition Screening Tool; MUST: Malnutrition Universal Screening Test; NRI: The Nutritional Risk Index; NRS: Nutritional Risk Screening 2002; NUTRIC: Nutrition Risk in the Critically Ill; ONS: Oral Nutritional Supplements; OPTN/SRTR: Organ Procurement and Transplantation Network/Scientific Registry of Transplant Recipients; PMI: Psoas Muscle mass Index; POD: Postoperative Day; QoL: Quality of Life; RFH-NPT: The Royal Free Hospital-Nutritional Prioritizing Tool; RFMS: Rectus Femoris Muscle Stiffness; RYGB: Roux-en-Y gastric bypass; SGA: Subjective Global Assessment; SICU: Surgical Intensive Care Unit; SMA: Skeletal Muscle Area; SMI: Skeletal Muscle Index; SNAQ: Simplified Nutritional Appetite Questionnaire; SO: Sarcopenic Obesity; SPPB: Short Physical Performance Battery; TSF: Triceps Skinfold Thickness; TUG: Timed-Up-and-Go test.
